# Pyrosequencing of supra- and subgingival biofilms from inflamed peri-implant and periodontal sites

**DOI:** 10.1186/1472-6831-14-157

**Published:** 2014-12-17

**Authors:** Simone Schaumann, Ingmar Staufenbiel, Ralph Scherer, Markus Schilhabel, Andreas Winkel, Sascha Nico Stumpp, Jörg Eberhard, Meike Stiesch

**Affiliations:** Department of Prosthetic Dentistry and Biomedical Materials Science, Hannover Medical School, Hannover, Germany; Department of Conservative Dentistry, Periodontology and Preventive Dentistry, Hannover Medical School, Hannover, Germany; Institute for Biometry, Hannover Medical School, Hannover, Germany; Institute of Clinical Molecular Biology, Christian-Albrechts-University Kiel, Kiel, Germany; Peri-implant and Oral Infections, Department of Prosthetic Dentistry and Biomedical Materials Science, Hannover Medical School, Carl-Neuberg-Strasse 1, 30625 Hannover, Germany

**Keywords:** Deep-sequencing, 16S rRNA sequencing, Diseased peri-implant tissues, Diseased periodontal tissues, Supragingival plaque, Subgingival plaque, Biofilm, Microbiology

## Abstract

**Background:**

To investigate the microbial composition of biofilms at inflamed peri-implant and periodontal tissues in the same subject, using 16S rRNA sequencing.

**Methods:**

Supra- and submucosal, and supra- and subgingival plaque samples were collected from 7 subjects suffering from diseased peri-implant and periodontal tissues. Bacterial DNA was isolated and 16S rRNA genes were amplified, sequenced and aligned for the identification of bacterial genera.

**Results:**

43734 chimera-depleted, denoised sequences were identified, corresponding to 1 phylum, 8 classes, 10 orders, 44 families and 150 genera. The most abundant families or genera found in supramucosal or supragingival plaque were *Streptoccocaceae, Rothia* and *Porphyromonas.* In submucosal plaque, the most abundant family or genera found were *Rothia, Streptococcaceae* and *Porphyromonas* on implants. The most abundant subgingival bacteria on teeth were *Prevotella, Streptococcaceae,* and *TG5.* The number of sequences found for the genera *Tannerella* and *Aggregatibacter* on implants differed significantly between supra- and submucosal locations before multiple testing. The analyses demonstrated no significant differences between microbiomes on implants and teeth in supra- or submucosal and supra- or subgingival biofilms.

**Conclusion:**

Diseased peri-implant and periodontal tissues in the same subject share similiar bacterial genera and based on the analysis of taxa on a genus level biofilm compositions may not account for the potentially distinct pathologies at implants or teeth.

**Electronic supplementary material:**

The online version of this article (doi:10.1186/1472-6831-14-157) contains supplementary material, which is available to authorized users.

## Background

Dental implants are commonly used to replace missing teeth in partially edentulous or edentulous patients. Inflammation of the peri-implant soft and hard tissue is the most frequent adverse event and may compromise the long-term stability of osseointegrated implants. While peri-implant mucositis affectes only soft tissues, peri-implantitis also involves the supporting bone. The prevalence of peri-implantitis during 5–10 years after successful osseointegration seems to be of the order of 10% of implants and 20% of patients [1].

Accepted risk factors for peri-implant related diseases are poor oral hygiene, a history of periodontitis and cigarette smoking [2]. Biofilms have been described in detail by using hybridization techniques in peri-implantitis [3–6] and recently by high-throughput sequencing techniques in failing implants [7–9]. Supra- and submucosal biofilms on implants in individual subjects have not been described by using high-throughput sequencing techniques, although it has been shown that the composition of supragingival biofims significantly affects subgingival biofilm formation [10–12]. In consequence, supramucosal biofilms may also determine the composition of the submucosal microflora. The diverse surface properties (chemical composition, surface roughness, surface free energy) and tissue architecture at implants and teeth may affect bacterial adhesion and growth of biofilms as well [13] and may account for the proposed differences in inflammatory response at implants and teeth [14].

Therefore the aim of the following study was to further characterise the microbial composition of supra- and submucosal, repectively supra- and subgingival plaques at diseased implants and teeth.

## Methods

### Subject selection

Subjects included in the study had at least ≥30% sites with PD ≥4 mm and evident radiographic bone loss. All patients were partially edentulous (not fewer than 8 teeth), with at least 1 functioning oral implant restored with crowns or prostheses. Inclusion criteria were: (A) one implant and teeth showing signs of active inflammation (tissue with manifest signs of inflammation (redness and swelling), bleeding on probing (BOP) and pocket depth (PD) ≥ 4 mm in at least one site and evidence of radiographic bone loss), (B) implants had to be functioning for at least 1 year. Exclusion criteria were: (A) any peri-implant or periodontal treatment 6 months before sampling. (B) systemic diseases such as diabetes mellitus, (C) smoking, (D) antibiotic or immunosuppressant medication within the previous 3 months.

A comprehensive medical history was recorded, followed by clinical and radiographic examination. Informed consent was obtained and the study was approved by the local Ethics committee of Hannover Medical School (no. 4348).

### Clinical examination

Two experienced dentists examined all subjects. Pocket depth was measured using a pressure calibrated periodontal probe (Hawe Click-Probe, Kerr Hawe SA, Bioggio, Switzerland). Probing depth was measured to the nearest millimeter on the scale. Bleeding on probing was assessed after probing using a dichotomous measure. All measurements were performed on 4 sites of all implants and teeth. Plaque deposits were recorded (presence/absence) without staining, using a modified Approximal Plaque Index (API) [15].

### Sample collection

In each subject, the implant and the tooth with the deepest depths were chosen for plaque collection. After isolating the sampling area with cotton rolls and gentle drying with an air syringe, 2 sterile endodontic paper points (Absorbent Paper Points, VDW GmbH, Munich, Germany) were used supramucosally or supragingivally to collect the biofilms. Subsequently, the residual supramucosal and supragingival plaques were completely removed with a dental scaler. Two sterile paper points were then placed submucosally or subgingivally. The samples were pooled separately for every implant, tooth and location and were placed in 2 ml cryotubes (Eppendorf, Hamburg, Germany) and frozen immediately at -80°C before processing.

### DNA extraction and sequencing

#### DNA isolation

Paper points used for sampling were treated with 360 μl lysozyme solution for 30 min at 37°C (20 mg/ml lysozyme, 20 mM TrisHCl, 2 mM EDTA, 1.2% Triton X100, pH 8.00), followed by proteinase K digestion for 30 min at 56°C in 400 μl buffer AL (Qiagen, Hilden, Germany). Enzymes were inactivated by heating to 95°C for 15 min. Sterile 0.5 mm glass beads (Roth, Karlsruhe, Germany) were added and bacterial cells were disrupted by vigorous shaking (6500 rpm, 3 x 20s, 15s break) with a Precellys 24 bead mill (Bertin Technologies, Montigny-le-Bretonneux, France). Subsequently, total DNA was purified with the QIAamp DNA Mini Kit (Qiagen, Hilden, Germany) according to the manufacturer’s protocol for gram-positive bacteria (QIAamp® DNA Mini and Blood Mini Handbook, Third Edition, Appendix D).

### 16S rDNA amplification and sample preparation

From each sample, an approximately 550 bp fragment of the 16S rRNA gene was amplified using the broad range primers 27f (5’-AGAGTTTGATCMTGGCTCAG-3´) and 521r (5’-ACCGCGGCTGCTGGCAC-3’; both Eurogentec, Seraing, Belgium). The primers targeted conserved DNA sequences flanking the V1 and V3 hypervariable regions within the 16S rRNA gene. PCR was performed on a TProfessional thermocycler (Biometra, Göttingen, Germany) in a total reaction volume of 50 μl. The PCR mix contained approximately 20 ng of template DNA, 200 nM of each primer, 1x PCR buffer (including 1.5 mM magnesium chloride; Qiagen, Hilden, Germany), 1.5U HotStar Taq polymerase (Qiagen, Hilden, Germany), 200 mM of each dNTP (Roth, Karlsruhe, Germany) and PCR-grade water (Roche, Penzberg, Germany). PCR conditions were as follows: Initial denaturation at 95°C for 15 min; 32 amplification cycles consisting of denaturation at 94°C for 1 min, annealing at 52°C for 40s, elongation at 72°C for 1 min; final extension at 72°C for 10 min. PCR reactions were separated on a 1.0% agarose gel (Agarose MP; AppliChem, Darmstadt, Germany) and purified using the QIAquick Gel Extraction Kit (Qiagen, Hilden, Germany). The purified amplicons of each sample were used as template for a second PCR step with the primer 27f-AdaB (5’-CCTATCCCCTGTGTGCCTTGGCAGTCTCAGAGAGTTTGATCMTGGCTCAG-3´) and an individual reverse primer 521r-MID_X (5’-CCATCTCATCCCTGCGTGTCTCCGACTCAGXXXXXXXXXXXACCGCGGCTGCTGGCAC-3’; XXXXXXXXXXX = unique MID-tag) containing a unique Multiplex-Identifier (MID) barcode sequence. Amplification chemistry was the same as described above, however, 100 ng of template DNA were used per reaction, the annealing temperature was raised to 67°C and the cycle number was reduced to 15. PCR reaction products were purified by agarose gel electrophoresis and extracted as described before. The DNA concentrations were determined using the AccuBlue™ High Sensitivity dsDNA Quantitation Kit (Biotium, Hayward, USA) in combination with a BioTekSynergy II fluorescence reader (BioTek, Bad Friedrichshall, Germany). Subsequently, the samples were mixed in an equimolar ratio and further processed according to the manufacturer’s instruction for the Titanium Library Preparation Kit (Roche, Penzberg, Germany). Pyrosequencing was performed on a GS FLX sequencer (Roche, Penzberg, Germany).

### Bioinformatics

#### Sequence processing

Qiime software version 1.6 [16] was used for preprocessing, the identification of operational taxonomic units (OTU), the taxonomic assignment and the community structure comparisons. In the preprocessing step, every 454-read was removed if (a) the number of base pairs was < 200 or > 550, (b) the quality score was < 25, (c) the number of ambiguous bases was > 6, (d) there was a primer mismatch, (e) the number of errors in barcode were > 1.5, or (f) a homopolymer run was > 6. In addition to these quality filtering steps, a denoising step of the sequences was performed [17] with the “denoise_wrapper”-script in qiime. Chimeric sequences were removed using ChimeraSlayer with the qiime default settings after OTU-picking and taxonomic assignment.

### OTU assignment and taxonomic classification

The sequences were assigned to OTUs with the uclust method in qiime with a similarity threshold of 0.97, which corresponds to genus level OTUs. For the following taxonomic assignment, we used the blast method in qiime with the greengenes 12_10 release with 97% OTUs as the reference database. In addition, genera were categorized according to their Gram staining based on Bergey’s Manual of Systematic Bacteriology.

### Statistical analyses

The OTU-table created by qiime after denoising and chimera checking was imported into the statistical programming language R [18] using the Bioconductor [19] package phyloseq [20]. The following graphical analyses were also performed using the phyloseq package and were created for (a) the whole data set, (b) the implant subset and (c) the tooth subset. The taxonomic rank used for the following analyses was the genus level. First, heat maps for the 50 most abundant bacteria were created. Second, Principal Coordinate Analyses (PCoA) of UniFac distances were calculated and plotted. The inferential statistical analysis was calculated with the Bioconductor package edgeR [21]. Therefore log Fold-Changes and corresponding multiplicity-adjusted p values were estimated from separate generalized linear models for every genus with patient as covariate and considering the paired design character. Biodiversity was calculated using the Shannon-Diversity Index [22].

## Results

### Clinical data

Seven subjects (2 males, 5 females, mean age 60.1 ± 9.8 years) were eligible for the study between August and October 2010 at Hannover Medical School, Department of Prosthetic Dentistry and Biomedical Materials Science. Individual data and full-mouth scorings of all patients are summarized in Table 1. All implants investigated had been functioning for an average of 11.6 ± 5.5 years. Clinical signs of inflammation were apparent at investigated implants (PD 4.9 ± 1.2 mm, BOP 39.9 ± 34.9 %) and teeth (PD 4.1 ± 1.2 mm, BOP 35.7 ± 31.8%). Differences between the clinical recordings at implants and teeth were not significant (Table 1).

**Table 1:** **Subject characteristics**

Study population	
Number of patients	7
Gender (male/female)	2/5
Age (years)	60.1 ± 9.8
Implant longevity (years)	11.6 ± 5.6
Number of Implants per patient (n)	4.7 ± 3.6
Number of remaining teeth per patient (n)	16.7 ± 7.3
Full-mouth scores	
Plaque index, API (%)	61.3 ±28.8
BOP (%)	22.1 ± 16.2
Number of periodontitis affected teeth per patient (%)	68.1 ± 15.5
Scores at sampled sites	
Implants	
Plaque index (%)	35.7 ± 37.8
BOP (%)	39.3 ± 34.9
PD (mm)	5.0 ± 1.3
Teeth	
Plaque index (%)	28.6 ± 39.3
BOP (%)	35.7 ± 31.8
PD (mm)	4.1 ± 1.3

Data are presented as means and standard deviations.

API, Approximal Plaque Index; BOP, bleeding on probing; PD, probing depths.

### Supra- and subgingival microbiomes

28 supra- and subgingival samples from 7 patients were analyzed and yielded a total of 43734 chimera-depleted, denoised sequences representing 1 phylum, 8 classes, 10 orders, 44 families and 150 genera (Additional file 1). On implants, these sequences represented the families *Porphyromonadaceae, Lachnospiraceae, Streptococcaceae* and genera *Rothia, Actinomyces, Paenibacillus, Microbacterium, Pseudoramibacter, Leptotrichia, Parascardovia, Tannerella, Granulicatella, Tessaracoccus, Clostridium, Aeromonadales, Veillonella, Capnocytophaga, Prevotella, TG5, Fusobacterium, Exiguobacterium, Enterococcus, Porphyromonas, Streptococcus* at implants. *On* teeth, the sequences represented the families *Coriobacteriaceae, Rs-045, Veillonellaceae, Neisseriaceae*, and the genera *Mogibacterium, Porphyromonas, Tannerella, Aggregatibacter, Treponema, Capnocytophaga, Lactococcus, Granulicatella, Enterococcus, Exiguobacterium, Atopobium, Veillonella*. On implants and teeth, the above-mentioned bacteria accounted for > 90% of all sequences.

**Figure 1:**
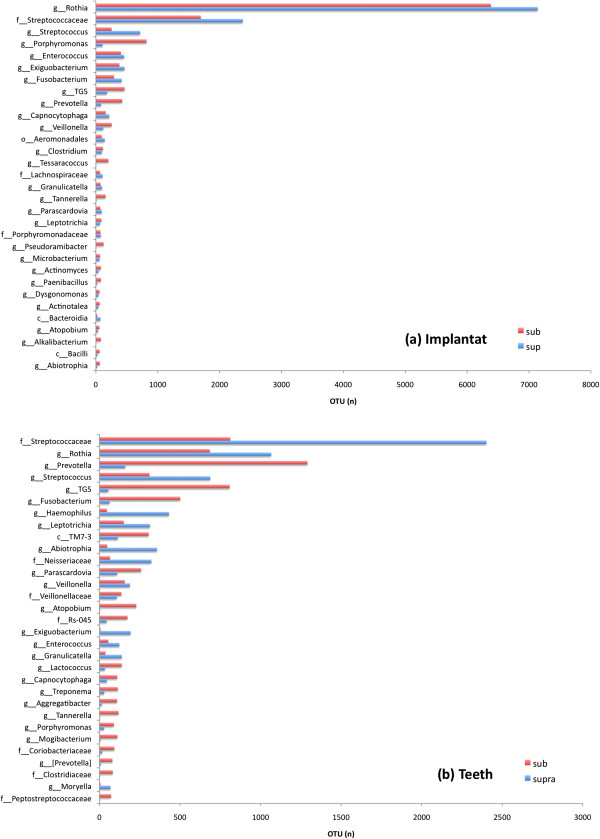
**Detection frequency of taxa found in inflamed peri-implant and periodontal sites. (a)** Distribution of taxa in supra- and submucosal biofilms from inflamed implants and **(b)** taxa in supra- and subgingival biofilms of teeth affected by periodontitis. The listed genera **(g)**, families **(f)** and classes **(c)** represents 90% of all sequences found.

In supramucosal or supragingival plaques on implants and teeth, the most abundant taxa were *Streptococcacea, Rothia,* and *Porphyromonas*. In submucosal plaques at implants, the most abundant taxa found were *Rothia, Streptococcaceae* and *Porphyromonas*. The most abundant subgingival bacteria on teeth were *Prevotella, Streptococcaceae and TG5* (Figure 1a, b).

The statistical analysis showed significant differences between supra- and submucosal plaque on implants for the genus *Tannerella* (p = 0.0067) and nearly significant differences for the genus *Aggregatibacter* (p = 0.056). After correction for multiple testing, these differences were no longer significant.

### Gram stain categories

**Figure 2:**
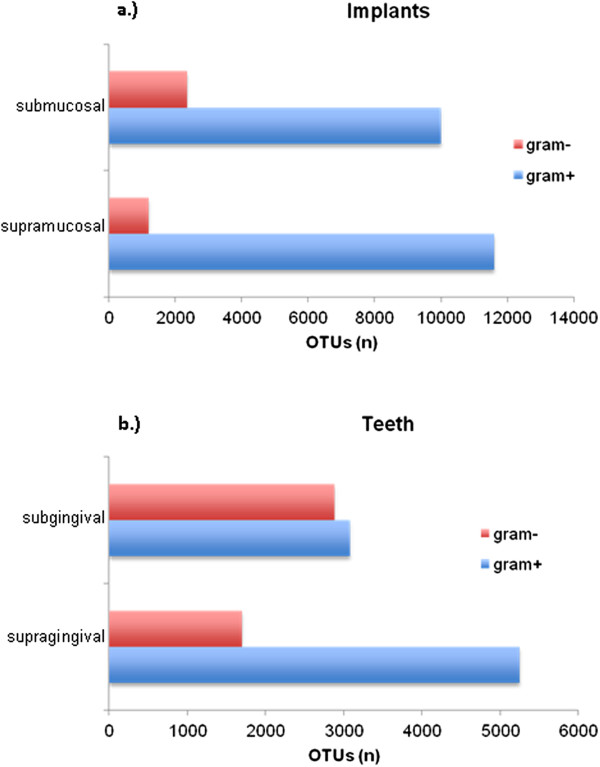
**The identified taxa were classified according to their Gram staining characteristics.** The bars represent the cumulative number of OTUs in supra- and submucosal areas at implants **(a)** and in supra- and subgingival areas at teeth **(b)**.

The Gram stain categories on implants and teeth are presented in Figure 2a and b. In general, Gram-positive bacteria were more prevalent than Gram-negative bacteria in all samples. On implants, Gram-positive bacteria were predominately found in supra- and submucosal samples. In supragingival samples of teeth, Gram-positive bacteria were more frequent than Gram-negative bacteria, but in subgingival plaque samples the abundances of Gram-positive and Gram-negative bacteria were similar. On implants and teeth, the number of Gram-negative bacteria were greater at submucosal and subgingival locations than at supramucosal and supragingival sites.

### Principal coordinate analysis (PCoA)

**Figure 3:**
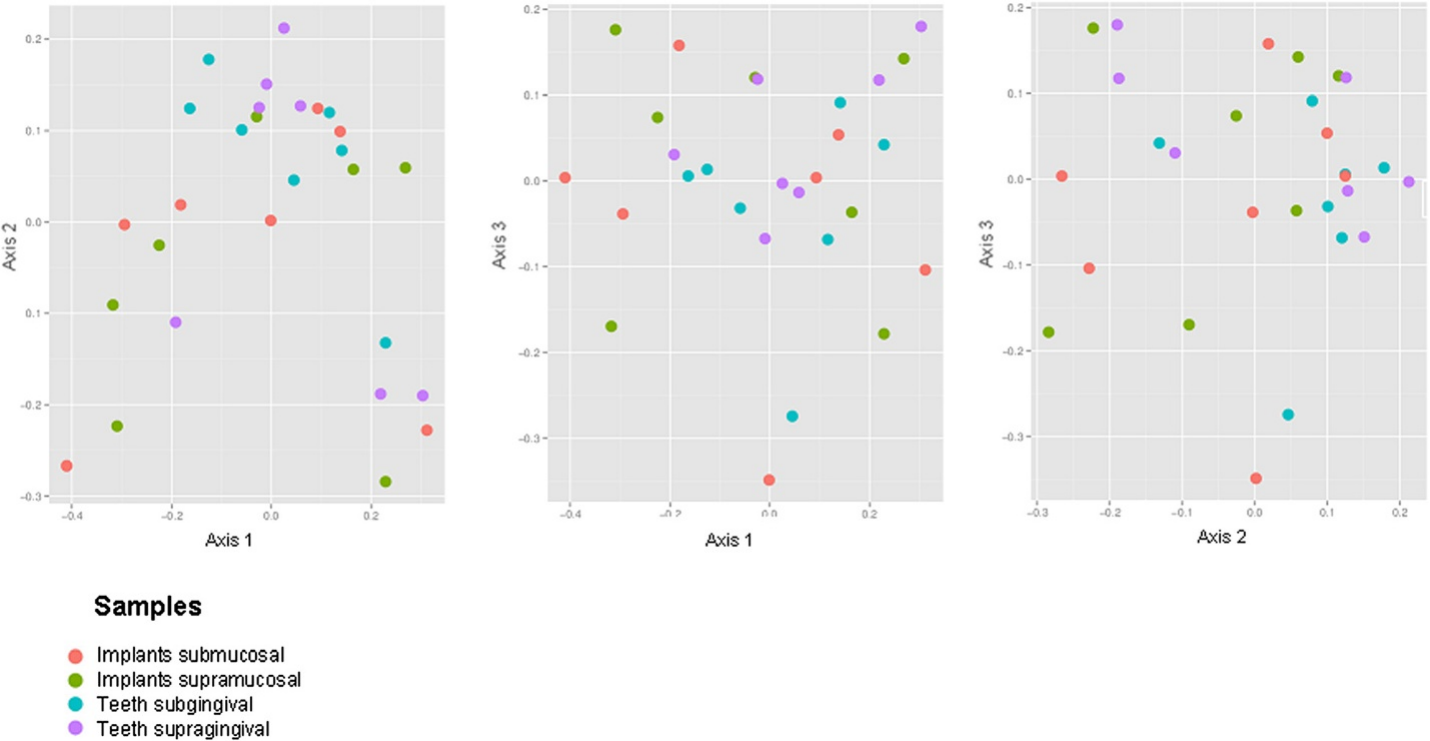
**Bacterial community structure at inflamed peri-implant and periodontal sites.** The panels show the Principal Co-ordinate Analysis of UniFac distances. There was no partitioning of the bacterial communities associated with implants or teeth (p > 0.01), as illustrated by the poorly graded distribution of dots representing the four sample areas of this study.

The Principal Coordinate Analysis (Figure 3) of weighted UniFac distances revealed no distinct partitioning of the bacterial communities associated with implants or teeth (p > 0.01).

### Heat map

**Figure 4:**
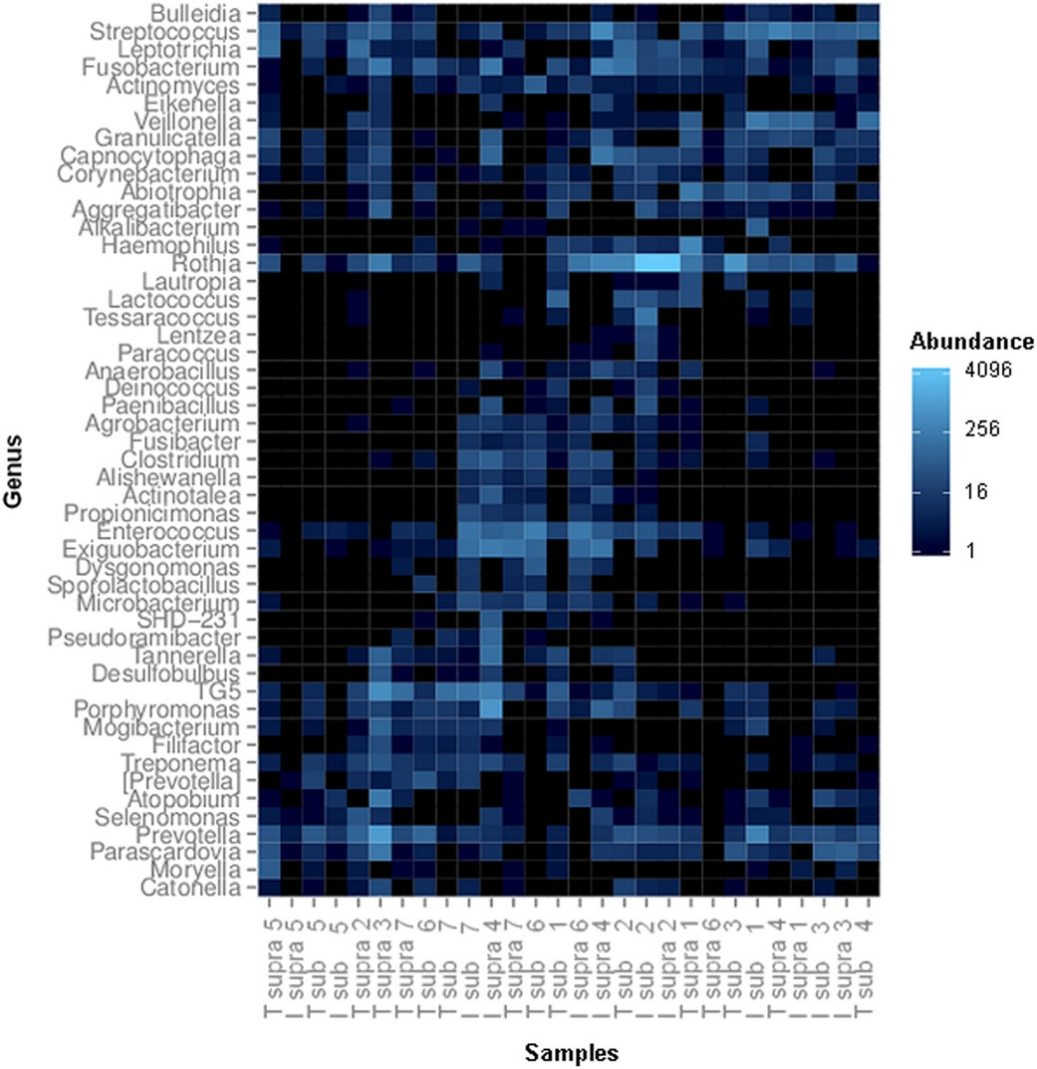
**Heat map presentation showing the abundances of the 50 most frequent genera in all samples.** Individual samples are depicted on the x-axis as tooth (T) or implant (I), the location supra (= supramucosal or supragingival) or sub (= submucosal or subgingival) and a number representing the patient. From this presentation, it is apparent that different locations within individual patients shared only minimal similarities in bacterial community compositions.

Data visualization was performed using a heat map display, where the relative abundances of the 50 most frequent genera are represented by different brightnesses (Figure 4). Samples from different locations within individual patients shared only minimal similarities in bacterial community compositions, as shown with hierarchical clustering of bacterial taxa in the heat map display. Communities from supramucosal locations at implants closely clustered with communities from submucosal locations at implants. In contrast, samples taken from supragingival plaque were less similar to subgingival plaque samples at teeth.

### Shannon diversity index

**Figure 5:**
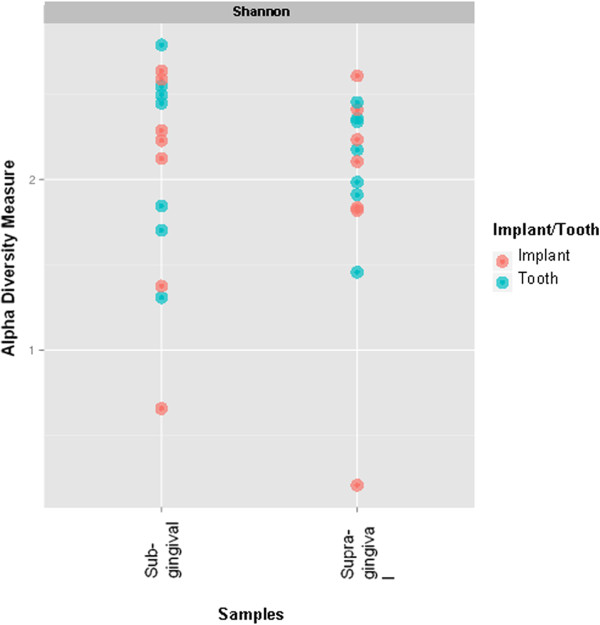
**The Shannon Diversity index was calculated for implants and teeth and showed that neither implants nor teeth demonstrated significant clustering of the diversity index of the sampling locations (blue and red dots).**

The Shannon Diversity index describes the biodiversity and considers the number of genera and their abundances [22]. Neither implants nor teeth demonstrated significant differences in the diversity index for supra- and submucosal locations at implants and supra- or subgingival locations at teeth (Figure 5).

## Discussion

The present study describes in detail the supra- and submucosal, and supra- and subgingival microbiomes of inflamed peri-implant and periodontal sites in single subjects using 16S rRNA gene-based pyrosequencing. The current study demonstrated (1) frequent occurrence of members of the genus *Rothia* and members of the family *Streptococcaceae* at implants and teeth, (2) no significant differences between the microbiomes of diseased implants and teeth affected by periodontitis, (3) no significant differences between supra- and submucosal, or supra- and subgingival microbiomes.

The current 16S rRNA approach was aimed to detect the comprehensive composition of bacteria located at two different sites at implants and teeth. In the present study, the sequencing lengths were limited to 550 bp and therefore annotations were restricted to the genus level, an established approach for the analysis of complex biofilms [23, 24]. In agreement with other current publications, the composition of microbiomes showed high inter-individual differences [8]. Prominent phylotypes at supra- and submucosal regions were *Rothia* and *Streptococcaceae*. Species belonging to the genus *Rothia* have been repeatedly described as members of oral communities [25–27], and have been associated with periodontal health [28, 29]. High levels of this genus have been reported at healthy implant sites as well [30]. Specific members of the genus *Rothia* have been recently shown to cause clinical infections such as septic arthritis, pneumonia, septicemia in renal transplant patients, arteriovenous infections, acute bronchitis and endocarditis [31] and - as a member of biofilms - has been associated with joint infections in orthopedics [32]. The virulence factors and the capacity of this genus to induce infections have been studied in vitro as well [33]. Our study also detected high frequencies of genera that have not been previously described as common oral inhabitants [34]. E.g. *Exiguobacterium* has been described as a bacterium colonizing marine habitats and sea food [35–37], ancient Siberian permafrost, Greenland glacial ice, and hot springs [38]. From the present study, it is unclear if this genus was accidentally incorporated by contamination [39] or if it was incorporated in oral plaques by food consumption., Food intake should therefore be accurately controlled or recorded in future studies.

All analyses in the present study indicated that the diversity of biofilms colonizing diseased implants was similar to biofilms colonizing teeth affected by periodontitis. In contrast, Kumar et al. [7] observed reduced diversity at implant sites than at diseased teeth and Koyanagi et al. [8] reported significantly higher diversity at implant sites than at diseased teeth. A partial explanation for these differences may be that the subjects were from different ethnic populations. It was hypothesized that diversity is an indicator of the complexity of a disease, whereas high diversity is associated with complex diseases.

In the present study, bacterial genera associated with diseased implants were not significantly different from communities associated with infected teeth in the same subject, which is in accordance with other publications [40–42] and demonstrated that the intraoral transmission of bacteria from one niche to the other is a feasible event. In contrast, with hybridization techniques the genus *Actinomyces* was the most dominant taxon found at teeth affected by periodontitis and diseased implants [3, 43], but was only found in low frequencies in the present study. Kumar et al. [7] used sequencing techniques and concluded that *Actinomyces* bacteria make up less than 5% of all sequences. The genera *Treponema and Tannerella* including species belonging to the red complex, as well as *Aggregatibacter,* were found in nearly similar frequencies at diseased implants and teeth affected by periodontitis; in contrast *Porphyromonas* was found more frequently at implants. The same observations were reported earlier by Cortelli et al. [44] but were not supported by other studies [7, 8]. Again, differences in the experimental design may account for these observations, e.g. Kumar et al. [7] investigated implants and teeth from different subjects.

In our study, the compositions of supra- or submucosal biofilms at implants were more similar than the supra- or subgingival biofilms at teeth, as demonstrated by the heat map analysis, which is in accordance to Ximenez-Fyvie et al. [43] who found identical genera in supra- and subgingival plaques of teeth affected by periodontitis. Utilizing DNA hybridization, Shibli et al. [3] also confirmed the similarities between biofilms at supra- and submucosal locations at implants.

At implant sites, the microbial composition was mainly composed of Gram-positive taxa. At teeth, Gram-positive taxa were also more frequent than Gram-negative taxa, but at much lower ratios. These differences between supra- and submucosal locations were not obvious on discrimination of sequenced genera, but became obvious using Gram characteristics. These data are partially in contrast to data reported by Kumar et al. [7], who stated that peri-implantitis of failing implants is a predominantly Gram-negative disease.

## Conclusions

The present study using 16S rRNA sequencing techniques complemented the knowledge of the composition of supra- and submucosal, and supra- and subgingal biofilms. Based on the limitations of the study and the analysis on a genus level significant differences in the biofilm composition of diseased peri-implant and periodontal tissues were not observed.

## Electronic supplementary material

Additional file 1: **16S rRNA gene sequences.** (ZIP 965 KB)

## Abbreviations

API: Approximal Plaque Index
BOP: Bleeding on probing
Bp: Base pair
DNA: Desoxyribonucleic acid
dNTP: Desoxynucleotide triphosphate
min.: Minute
ml: Milliliter
mm: Millimeter
no: Number
OUT: Operational taxonomic units
PCoA: Principal Coodinate Analyses
PCR: Polymerase Chain Reaction
PD: Pocket depth
rRNA: Ribosomal ribonucleic acid.

## References

[1] Mombelli A, Muller N, Cionca N (2012). The epidemiology of peri-implantitis. Clin Oral Implants Res.

[2] Heitz-Mayfield LJ (2008). Peri-implant diseases: diagnosis and risk indicators. J Clin Periodontol.

[3] Shibli JA, Melo L, Ferrari DS, Figueiredo LC, Faveri M, Feres M (2008). Composition of supra- and subgingival biofilm of subjects with healthy and diseased implants. Clin Oral Implants Res.

[4] Persson GR, Samuelsson E, Lindahl C, Renvert S (2010). Mechanical non-surgical treatment of peri-implantitis: a single-blinded randomized longitudinal clinical study. II. Microbiological results. J Clin Periodontol.

[5] Maximo MB, de Mendonca AC, Renata Santos V, Figueiredo LC, Feres M, Duarte PM (2009). Short-term clinical and microbiological evaluations of peri-implant diseases before and after mechanical anti-infective therapies. Clin Oral Implants Res.

[6] Salcetti JM, Moriarty JD, Cooper LF, Smith FW, Collins JG, Socransky SS, Offenbacher S (1997). The clinical, microbial, and host response characteristics of the failing implant. Int J Oral Maxillofac Implants.

[7] Kumar PS, Mason MR, Brooker MR, O'Brien K (2012). Pyrosequencing reveals unique microbial signatures associated with healthy and failing dental implants. J Clin Periodontol.

[8] Koyanagi T, Sakamoto M, Takeuchi Y, Maruyama N, Ohkuma M, Izumi Y (2013). Comprehensive microbiological findings in peri-implantitis and periodontitis. J Clin Periodontol.

[9] Dabdoub SM, Tsigarida AA, Kumar PS (2013). Patient-specific analysis of periodontal and peri-implant microbiomes. J Dent Res.

[10] Hellstrom MK, Ramberg P, Krok L, Lindhe J (1996). The effect of supragingival plaque control on the subgingival microflora in human periodontitis. J Clin Periodontol.

[11] Socransky SS, Haffajee AD (2002). Dental biofilms: difficult therapeutic targets. Periodontol 2000.

[12] Tezal M, Scannapieco FA, Wactawski-Wende J, Grossi SG, Genco RJ (2006). Supragingival plaque may modify the effects of subgingival bacteria on attachment loss. J Periodontol.

[13] Teughels W, Van Assche N, Sliepen I, Quirynen M (2006). Effect of material characteristics and/or surface topography on biofilm development. Clin Oral Implants Res.

[14] Heitz-Mayfield LJ, Lang NP (2010). Comparative biology of chronic and aggressive periodontitis vs. peri-implantitis. Periodontol 2000.

[15] Lange DE, Plagmann H-C, Eenboom A, Promesberger A (1977). Klinische Bewertungsverfahren zur Objektivierung der Mundhygiene. Dtsch zahnärztl Z.

[16] Caporaso JG, Kuczynski J, Stombaugh J, Bittinger K, Bushman FD, Costello EK, Fierer N, Pena AG, Goodrich JK, Gordon JI, Huttley GA, Kelley ST, Knights D, Koenig JE, Ley RE, Lozupone CA, McDonald D, Muegge BD, Pirrung M, Reeder J, Sevinsky JR, Turnbaugh PJ, Walters WA, Widmann J, Yatsunenko T, Zaneveld J, Knight R (2010). QIIME allows analysis of high-throughput community sequencing data. Nat Methods.

[17] Reeder J, Knight R (2010). Rapidly denoising pyrosequencing amplicon reads by exploiting rank-abundance distributions. Nat Methods.

[18] R Core Team (2013). R: A language and environment for statistical computing.

[19] Gentleman RC, Carey VJ, Bates DM, Bolstad B, Dettling M, Dudoit S, Ellis B, Gautier L, Ge Y, Gentry J, Hornik K, Hothorn T, Huber W, Iacus S, Irizarry R, Leisch F, Li C, Maechler M, Rossini AJ, Sawitzki G, Smith C, Smyth G, Tierney L, Yang JY, Zhang J (2004). Bioconductor: open software development for computational biology and bioinformatics. Genome Biol.

[20] McMurdie PJ, Holmes S (2013). phyloseq: an R package for reproducible interactive analysis and graphics of microbiome census data. PLoS One.

[21] Robinson MD, McCarthy DJ, Smyth GK (2010). edgeR: a Bioconductor package for differential expression analysis of digital gene expression data. Bioinformatics.

[22] Shannon CE (1963). The mathematical theory of communication. MD Comput 1997.

[23] Bizzarro S, Loos BG, Laine ML, Crielaard W, Zaura E (2013). Subgingival microbiome in smokers and non-smokers in periodontitis: an exploratory study using traditional targeted techniques and a next-generation sequencing. J Clin Periodontol.

[24] Hu YJ, Wang Q, Jiang YT, Ma R, Xia WW, Tang ZS, Liu Z, Liang JP, Huang ZW (2013). Characterization of oral bacterial diversity of irradiated patients by high-throughput sequencing. Int J Oral Sci.

[25] Tanner AC, Haffer C, Bratthall GT, Visconti RA, Socransky SS (1979). A study of the bacteria associated with advancing periodontitis in man. J Clin Periodontol.

[26] Lesher RJ, Gerencser VF, Morrison DJ (1977). Presence of Rothia dentocariosa strain 477 serotype 2 in gingiva of patients with inflammatory periodontal disease. J Dent Res.

[27] Segata N, Haake SK, Mannon P, Lemon KP, Waldron L, Gevers D, Huttenhower C, Izard J (2012). Composition of the adult digestive tract bacterial microbiome based on seven mouth surfaces, tonsils, throat and stool samples. Genome Biol.

[28] Kistler JO, Booth V, Bradshaw DJ, Wade WG (2013). Bacterial community development in experimental gingivitis. PLoS One.

[29] Abusleme L, Dupuy AK, Dutzan N, Silva N, Burleson JA, Strausbaugh LD, Gamonal J, Diaz PI (2013). The subgingival microbiome in health and periodontitis and its relationship with community biomass and inflammation. ISME J.

[30] da Silva ES, Feres M, Figueiredo LC, Shibli JA, Ramiro FS, Faveri M (2014). Microbiological diversity of peri-implantitis biofilm by Sanger sequencing. Clin Oral Implants Res.

[31] Shakoor S, Fasih N, Jabeen K, Jamil B (2011). Rothia dentocariosa endocarditis with mitral valve prolapse: case report and brief review. Infection.

[32] Trivedi MN, Malhotra P (2013). Rothia prosthetic knee joint infection. J Microbiol Immunol Infect.

[33] Kataoka H, Taniguchi M, Fukamachi H, Arimoto T, Morisaki H, Kuwata H (2014). Rothia dentocariosa induces TNF-alpha production in a TLR2-dependent manner. Pathog Dis.

[34] Chen T, Yu WH, Izard J, Baranova OV, Lakshmanan A, Dewhirst FE (2010). The Human Oral Microbiome Database: a web accessible resource for investigating oral microbe taxonomic and genomic information. Database (Oxford).

[35] Inbakandan D, Murthy PS, Venkatesan R, Khan SA (2010). 16S rDNA sequence analysis of culturable marine biofilm forming bacteria from a ship's hull. Biofouling.

[36] Wen W, Wang S, Zhou X, Fang B (2013). Central carbon metabolism in marine bacteria examined with a simplified assay for dehydrogenases. Appl Biochem Biotechnol.

[37] Yang J, Wang C, Wu J, Liu L, Zhang G, Feng J (2014). Characterization of a multi-resistant mosaic plasmid from a fish farm sediment Exiguobacterium sp. isolate reveals aggregation of functional clinically-associated antibiotic resistance genes. Appl Environ Microbiol.

[38] Vishnivetskaya TA, Kathariou S, Tiedje JM (2009). The Exiguobacterium genus: biodiversity and biogeography. Extremophiles.

[39] van der Horst J, Buijs MJ, Laine ML, Wismeijer D, Loos BG, Crielaard W, Zaura E (2013). Sterile paper points as a bacterial DNA-contamination source in microbiome profiles of clinical samples. J Dent.

[40] Sumida S, Ishihara K, Kishi M, Okuda K (2002). Transmission of periodontal disease-associated bacteria from teeth to osseointegrated implant regions. Int J Oral Maxillofac Implants.

[41] Quirynen M, Papaioannou W, van Steenberghe D (1996). Intraoral transmission and the colonization of oral hard surfaces. J Periodontol.

[42] Quirynen M, Vogels R, Peeters W, van Steenberghe D, Naert I, Haffajee A (2006). Dynamics of initial subgingival colonization of 'pristine' peri-implant pockets. Clin Oral Implants Res.

[43] Ximenez-Fyvie LA, Haffajee AD, Socransky SS (2000). Microbial composition of supra- and subgingival plaque in subjects with adult periodontitis. J Clin Periodontol.

[44] Cortelli SC, Cortelli JR, Romeiro RL, Costa FO, Aquino DR, Orzechowski PR, Araujo VC, Duarte PM (2013). Frequency of periodontal pathogens in equivalent peri-implant and periodontal clinical statuses. Arch Oral Biol.

[CR45] The pre-publication history for this paper can be accessed here:http://www.biomedcentral.com/1472-6831/14/157/prepub

